# Positive Retrospective SARS-CoV-2 Testing in a Case of Acute Respiratory Distress Syndrome of Unknown Etiology.

**DOI:** 10.1155/2021/5484239

**Published:** 2021-08-28

**Authors:** A. Burkett, S. McElwee, C. Margaroli, P. Bajpai, A. Elkholy, U. Manne, K. Wille, P. Benson

**Affiliations:** ^1^Department of Pathology, Division of Anatomic Pathology, University of Alabama at Birmingham, Birmingham, Alabama, USA; ^2^Department of Medicine, Division of Cardiovascular Disease, University of Alabama at Birmingham, Birmingham, Alabama, USA; ^3^Department of Medicine, Division of Pulmonary, Allergy & Critical Care Medicine, University of Alabama at Birmingham, Birmingham, Alabama, USA

## Abstract

In order to elucidate the cause of acute respiratory distress syndrome of unknown etiology in a pre-pandemic patient, molecular techniques were used for detection of SARS-CoV-2. We used a SARS-CoV-2 nucleocapsid protein immunofluorescence stain to retrospectively identify an individual with diffuse alveolar damage on autopsy histology who had negative respiratory virus panel results in February, 2020, in Birmingham, Alabama. In situ hybridization for SARS-CoV-2 RNA revealed evidence of widespread multiorgan SARS-CoV-2 infection. This death antecedes the first reported death of a State of Alabama resident diagnosed with SARS-CoV-2 by 26 days.

## 1. Introduction

Though the initial outbreak was described in Wuhan, China, with subsequent spread to the United States by January 2020, it has been assumed that the infection emerged months prior to being recognized by the World Health Organization [1]. The first case of COVID-19 in Alabama was reported on March 12, 2020 [2]. Prior to this report, the first reported death of an Alabama resident with COVID-19 occurred on March 25, 2020 [3]. To determine the cause of acute respiratory distress syndrome (ARDS), molecular techniques for the detection of SARS-CoV-2 were used on autopsy tissue.

## 2. Case Report

**Figure 1:**
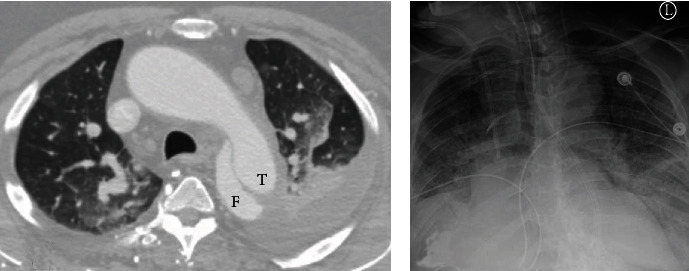
(a) CTA of chest (February 16, 2020) with Type B aortic dissection (T: true lumen; F: false lumen). (b) Chest X-ray (February 28, 2020) with bilateral pulmonary opacities and pleural effusions.

A 79-year-old male with hypertensive cardiovascular disease, heart failure, hyperlipidemia, and diabetes type 2 presented from an outside hospital on February 9, 2020, with acute chest pain and dyspnea. CT angiogram (CTA) showed a Type B dissection and ascending aortic aneurysm. He was transferred to our tertiary care hospital. Work-up revealed metabolic derangement, cardiomegaly with trace pericardial effusion, pleural effusions, and diffuse ground glass opacities suggestive of pulmonary edema. His hospital course was complicated by difficulties maintaining his oxygen saturation, requiring high flow nasal cannula; CTA on February 16 demonstrated the Type B aortic dissection was suggestive of pulmonary embolism and showed ground glass attenuation of the bilateral aerated lung fields and consolidation of the left lower lobe (Figure 1(a)). The patient was advanced to BiPAP without improvement and was transferred to the cardiopulmonary critical care unit for hypoxic respiratory failure, requiring intubation on February 24, 2020. A bronchoscopy confirmed ARDS. A respiratory viral panel was negative for Influenza A (subtype H1, H3 and 2009 H1N1), Influenza B, RSV A and B, Parainfluenza 1, 2, 3, and 4, Human Metapneumovirus, Human Rhinovirus/Enterovirus, Adenovirus, and Coronavirus. On February 25, the patient had PEA arrest, but return of spontaneous circulation was achieved with chest compressions. Chest X-ray on February 28, 2020, showed bilateral pulmonary opacities and pleural effusions (Figure 1(b)). He continued to be unable to maintain oxygen saturation despite intubation and aggressive fluid removal. Death occurred on February 28, 2020.

**Figure 2:**
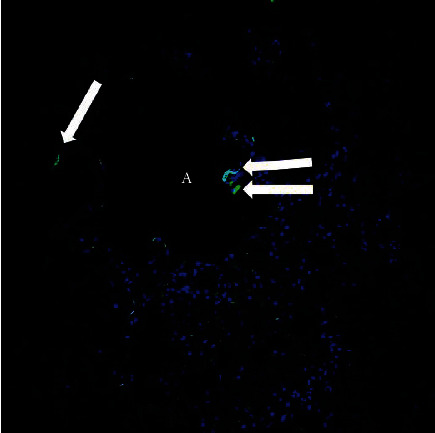
SARS-CoV-2 nucleocapsid protein immunofluorescence in lung. Arrows indicate green positive signal. A = alveolar space. Blue = DAPI nuclear stain.

**Figure 3:**
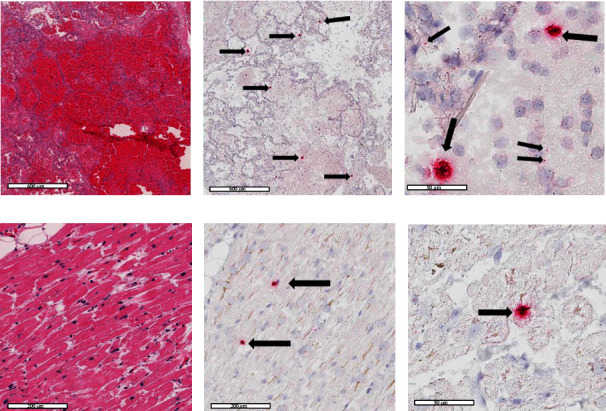
(a) Lung H&E 4x (scale bar: 600 *μ*m) with diffuse alveolar hemorrhage and alveolar fibrin. (b) SARS-CoV-2 ISH 4x (scale bar: 50 *μ*m). (c) SARS CoV-2 ISH 40x (scale bar: 50 *μ*m). (d) Heart H&E 20x E: Heart SARS-CoV-2 ISH 20x (scale bar: 200 *μ*m). (f) Heart SARS CoV-2 ISH 40x (scale bar: 50 *μ*m). Black arrows: positive antisense SA RS-CoV-2 RNA probe red signal.

**Figure 4:**
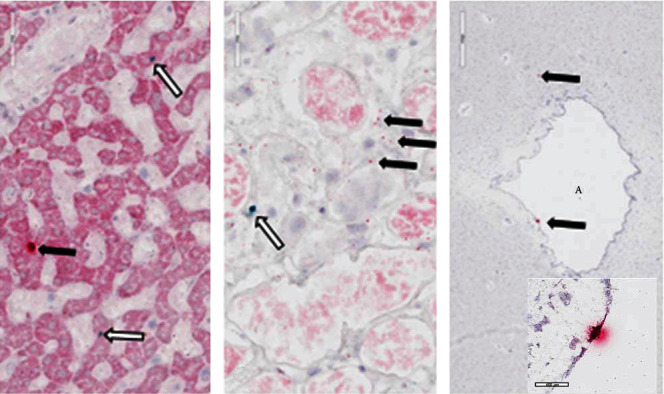
SARS-CoV-2 ISH of the liver (a) and kidney (b) (40x: scale bar, 60 *μ*m) and midbrain (c) (2x: scale bar, 600 *μ*m) and inset 40x (scale bar, 50 *μ*m). A: cerebral aqueduct. Black arrows: red signal (antisense strand RNA-replicating virus). White arrows: turquois signal (sense strand RNA-nonreplicating virus).

Written consent for autopsy was obtained from the next of kin including consent for diagnostic, research, and education use; however, no written consent has been obtained from the patient as there is no identifiable patient data included in this case report. Autopsy performed March 2, 2020, showed signs of hypertensive and atherosclerotic cardiovascular disease with severe cardiomegaly (650 g) and four chamber dilatation, nephrosclerosis, and diffuse hypertensive vascular changes within the brain. There was dilation of the aortic root and a Type B aortic dissection extending from the left subclavian artery without rupture. The lungs showed significant bilateral consolidation (combined lung weight of 1970 g) and pulmonary edema without evidence of a pulmonary thromboembolism. Lung histology showed diffuse intra-alveolar hemorrhage and fibrin, with numerous areas showing organizing fibrinous exudates with macrophages and occasional activated type II pneumocytes. Chronic bronchitis and emphysema were present. Immunofluorescence for SARS-CoV-2 nucleocapsid protein was focally positive on one lung section (Figure 2). In situ hybridization (ISH) for SARS-CoV-2 RNA was performed using RNA scope technology (Bio-Techne Corp., Minneapolis, MN). Two RNA probes were used, one for the sense strand of SARS-CoV-2 RNA (turquoise signal) and one for the antisense strand of SARS-CoV-2 RNA (red signal). ISH showed SARS-CoV-2 in the lungs, myocardium, liver, kidneys, and brain (Figures 3 and 4). Both ISH probes showed varying degrees of intensity. The lungs, heart, and brain showed predominantly red signal indicating replicating virus. Sections of the liver and kidney showed areas of turquoise signal indicating nonreplicating virus in addition to red replicating virus signal (Figure 4). Replicating virus signal was present in the ependymal lining cells and in the parenchyma of the midbrain (Figure 4(c)).

## 3. Discussion

Two reports used molecular techniques to show no earlier emergence of COVID-19 in California, USA [4], and Basel, Switzerland [5]. A more recent report successfully identified SARS-CoV-2 in a Canadian renal transplant patient whose death occurred on February 10, 2020 [6]. The case presented here is the first to retrospectively identify a case of lethal SARS-CoV-2 infection with clinical ARDS which predates the first recognized SARS-CoV-2-related death in Alabama by 26 days. Although earlier cases were later detected, the Center for Disease Control reported the first COVID-19 death in Washington State on February 29, 2020, one day later than the case we report here [7]. While the identification of a case demonstrates an earlier communal spread of the SARS-CoV-2 virus, additional retrospective studies are essential in determining the earliest regional and national transmission of the virus. The SARS-CoV-2 RNA ISH showed widespread involvement of multiple organs as would be expected in viremia.

## 4. Conclusion

Our report confirms the presence of SARS-CoV-2 in Alabama 13 days prior to its reported emergence in the state and precedes the first reported SARS-CoV-2 death in Alabama by 26 days. The widespread positive staining for SARS-CoV-2 RNA in the lungs, heart, liver, kidneys, and brain confirmed the disseminated nature of the infection and the cause of the patient's ARDS. Analysis of additional prepandemic autopsy cases may add to the understanding of epidemiology of COVID-19 and provide retrospective answers to clinical questions.

## Data Availability

The data supporting the results of the study are presented in the manuscript.
